# Management of fibrosis in neovascular age-related macular degeneration

**DOI:** 10.1007/s00417-025-06890-x

**Published:** 2025-10-22

**Authors:** Usha Chakravarthy, Alexander J. E. Foss, Georgios D. Panos, Tunde Peto, Tryfon Rotsos, SriniVas Sadda, Eduard De Cock, Theo Empeslidis

**Affiliations:** 1 Queen’s University Belfast, Institute of Clinical Science, Royal Victoria Hospital, Belfast, UK; 2 Department of Ophthalmology, Queen’s Medical Centre, University of Nottingham, Nottingham, UK; 3 Division of Ophthalmology and Visual Sciences, School of Medicine, Nottingham University Hospitals, Nottingham, UK; 4 Centre for Public Health, Queen’s University Belfast, Belfast, UK; 5 Department of Ophthalmology, Democritus University of Thrace, Alexandroupolis, Greece; 6 Doheny Image Reading Center, Doheny Eye Institute, David Geffen School of Medicine, University of California Los Angeles, Los Angeles, CA USA; 7 Boehringer Ingelheim International GmBH, Ingelheim am Rhein, Germany

**Keywords:** Age-related macular degeneration, Anti-VEGF, Fibrosis, Pathogenesis, Risk factors, Subretinal hyper-reflective material

## Abstract

Subretinal fibrosis is a common end-stage sequela of neovascular age-related macular degeneration (nAMD), and it is associated with poor long-term visual outcomes. The pathogenesis of subretinal fibrosis in nAMD is largely driven by epithelial–mesenchymal and endothelial–mesenchymal transition within the retinal pigment epithelium and endothelium of the choroidal circulation. Upregulation of vascular endothelial growth factor (VEGF) expression further contributes to the observed fibrovascular content and increased vascular permeability. There is a substantial need for direct therapeutic strategies for fibrosis in nAMD, including anti-fibrotic agents. Until direct treatment strategies are developed, the management of nAMD using anti-VEGF agents must be optimized. However, fibrosis can occur in some patients otherwise successfully treated with anti-VEGF therapy, resulting in the loss of previous visual acuity (VA) gains experienced after treatment initiation. This review summarizes the current understanding of the mechanisms driving fibrosis in nAMD, risk factors for fibrosis development, and limitations of current detection methods. Available evidence on how different factors relating to anti-VEGF therapy (e.g., specific agent, delays in administration, extended administration intervals, dosing or treatment regimen) and the early detection of nAMD impact the risk of fibrosis is also discussed. Lastly, insights into potential future directions for the management of fibrosis in nAMD, particularly the development of anti-fibrotic agents, are deliberated.

## Key Messages

*****What is known***:**

- Subretinal fibrosis is a common feature of late-stage neovascular age-related macular degeneration (nAMD) associated with poor long-term visual outcomes; current therapies may limit the severity of fibrosis but do not prevent its development

*****What is new***:**

- This review summarizes the current understanding of the mechanisms of fibrosis in nAMD, risk factors for fibrosis development, and limitations of current detection methods
- There are several risk factors associated with fibrosis development and anti-vascular endothelial growth factor therapies are the only treatment option for nAMD, and optimized use of such therapies can reduce the likelihood of fibrosis development in nAMD
- Reducing incident fibrosis can help preserve visual acuity (VA) gains, through minimizing treatment delays and instituting appropriate monitoring strategies to ensure timely re-treatment in the event of reactivation of the nAMD lesion

## Introduction

**Fig. 1:**
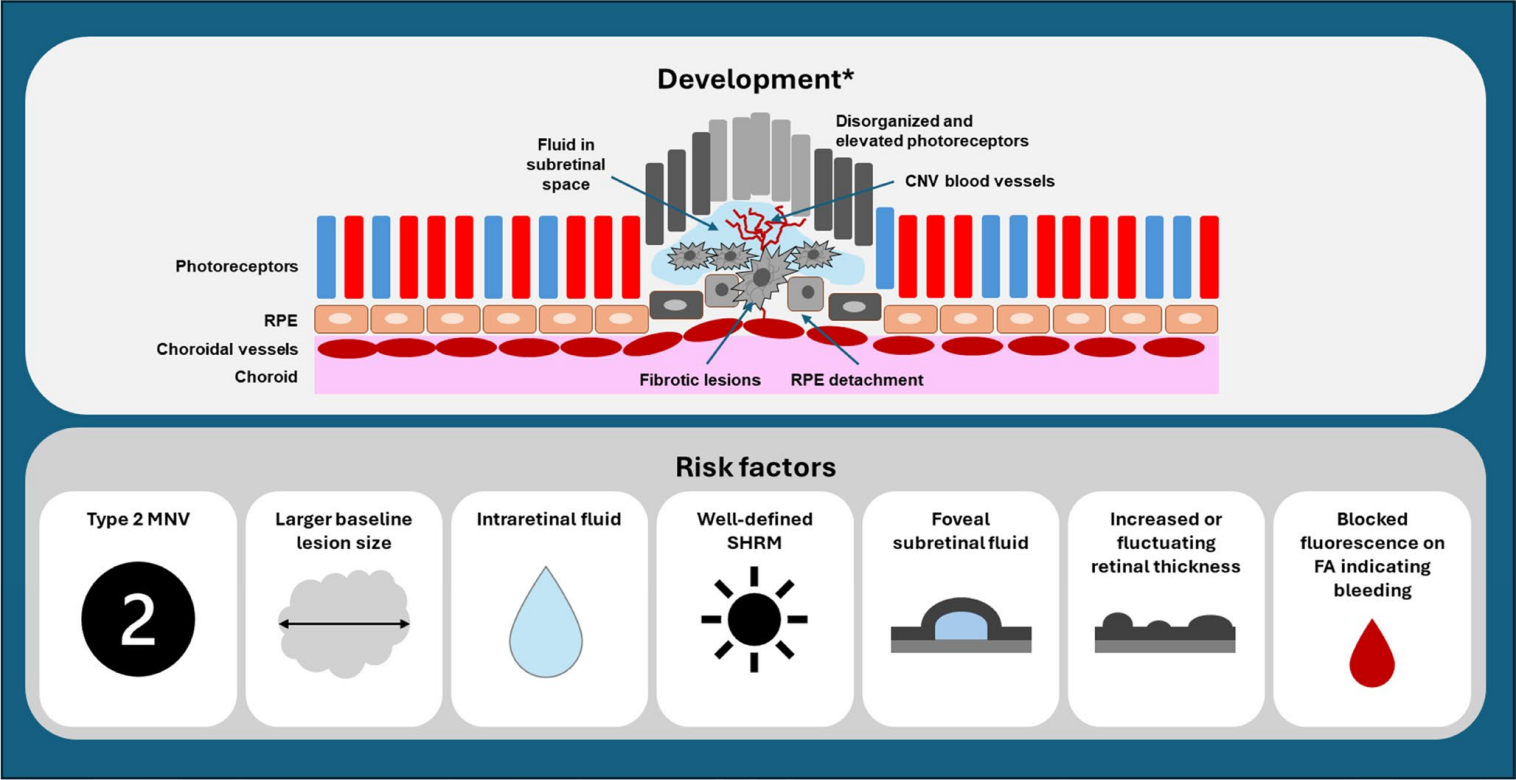
Pathophysiology and risk factors for retinal fibrosis in nAMD. *Upper panel of figure adapted from Shu DY, et al. Int J Mol Sci. 2020;21:4271. CNV choroidal neovascularization; FA fundus autofluorescence; MNV macular neovascularization; nAMD neovascular age-related macular degeneration; RPE retinal pigment epithelium; SHRM subretinal hyperreflective material

Neovascular age-related macular degeneration (nAMD) is characterized by the rapid onset of acute vision loss, resulting from pathological neovascularization from the choroid into the subretinal or retinal pigment epithelium (RPE) spaces [1, 2]. Fibrosis, sometimes termed a disciform scar [3], is a common end-stage sequela of nAMD associated with poor long-term visual outcomes [4]. Fibrosis in nAMD typically develops slowly, often over the course of several years, although it can develop over shorter timeframes (see Fig. 1) [4–6]. Therefore, the availability of suitable treatments for fibrosis would allow adequate time for intervention, but at present, no such therapy is available [7]. In patients who have had macular neovascularization (MNV) for 12 months or longer, fibrosis can occupy up to 30% of the area of an MNV lesion despite treatment with anti–vascular endothelial growth factor (anti-VEGF) therapy [8]. Subretinal fibrosis and the development of atrophy have both been identified as the main causes of at least 10-letter loss in VA [9–11]. Risk factors for fibrosis include classic (type 2) MNV, baseline lesion size (odds ratio 1.08; 95% confidence interval [CI] 1.08 − 1.14] per 1000 μm; *p* = 0.03), intraretinal fluid, well-defined subretinal hyper-reflective material (SHRM), foveal subretinal fluid, increased retinal thickness or fluctuations in retinal thickness, and blocked fluorescence on fluorescein angiography (FA) as an indication of bleeding (see Fig. 1) [6, 7, 11, 12]. On optical coherence tomography (OCT), the presence of SHRM, primarily comprising fibrovascular material and associated with a vascular network, may also be considered a biomarker for retinal fibrosis [13].

Upregulation of VEGF contributes to both the fibrovascular content and increased vascular permeability observed in nAMD [14]. The current standard of care for nAMD worldwide is anti-VEGF therapy, which may also reduce the occurrence of fibrosis over time [12, 15]. Fibrosis can occur in some patients otherwise successfully treated with anti-VEGF agents, resulting in loss of the VA gains experienced after treatment initiation [11, 16–18]. Following anti-VEGF therapy, MNV lesion sizes generally reduce, but areas of fibrosis may persist or even increase in size [8].

Several large, randomized studies (i.e., CATT, IVAN, HARBOR) have reported on the development of late fibrosis despite ongoing anti-VEGF therapy in 43.3–79.5% of patients after 2 years of treatment [5]; the high incidence of this late-stage complication constitutes a major clinical problem. In a *post hoc* analysis of the CATT and IVAN trials with 2 years of follow-up, the proportion of eyes with fibrosis increased from 7.8% (*n* = 135/1720) at baseline to 58.7% (*n* = 931/1586) at the final visit [6]. In eyes without fibrosis at baseline, 54.7% (*n* = 789/1443) developed fibrosis by the final visit [6]. Real-world prevalence rates seen in prospective or retrospective studies were wider than those observed in controlled clinical trials, both at treatment initiation and at subsequent time points during follow-up [5]. In a retrospective, single-center, observational study of 150 eyes of 144 patients, the proportion of eyes with fibrotic scarring significantly increased from 15.3% (*n* = 23/150) at baseline to 27.3% (*n* = 41/150) after 1 year of follow‑up (*p* < 0.001) [19]. In a systematic review and meta-analysis that included studies with follow‑up out to 60 months in nAMD managed with anti-VEGF therapies, the prevalence of fibrosis at baseline, 12, 24, and 60 months was 13%, 32%, 36%, and 56%, respectively [11]. In the IVAN trial, after up to 7 years of follow-up, hyper-reflective material (HRM), a proxy for fibrosis, graded on OCT was present in 78.8% of study eyes (*n* = 360/457), and its location was primarily sub-RPE (71.4%) and subretinal (59.4%), but rarely intraretinal (1.7%) [20]. The prevalence of fibrosis after 10 years of treatment with anti-VEGF agents is approximately 40% [12], highlighting the need for additional therapeutic options. However, until novel treatments targeting the drivers of fibrosis are developed, nAMD management using anti-VEGF agents must be optimized.

In this article, we review the mechanisms of fibrosis in nAMD, detection methods, risk factors for development, and existing treatment strategies. We summarize available evidence on how different factors relating to anti-VEGF therapy (e.g., specific agent, delays or extended intervals, dosing or treatment regimen) and early nAMD detection impact fibrosis risk. Finally, we consider potential future directions to reduce the risk and impact of fibrosis in this patient population.

## Defining and detecting fibrosis in nAMD

In nAMD, fibrosis exacerbates the healing response of the neovascular membranes, resulting in scarring. This process is driven by chronic inflammation, cytoskeletal changes, RPE migration, and dedifferentiation, driving cells towards a mesenchymal phenotype [5, 21]. Subsequently, recruitment of fibroblasts and the deposition of collagen results in a scar comprising endothelial cells, RPE, macrophages, fibroblasts, and extracellular matrix materials [5].

Accurate detection and quantification of fibrosis are essential in optimizing treatment outcomes, but the lack of a consensus definition of fibrosis in nAMD makes this challenging [5], and ongoing efforts to develop a new multimodal classification system are still pending [5]. Each imaging modality characterizes fibrosis differently; for example, fibrosis is visible as white scar tissue using color fundus photography (CFP) but appears as SHRM using OCT. Additionally, SHRM does not always indicate fibrosis; therefore, poorly defined fibrinous areas (also called undefined lesions) must be distinguished from well-defined lesions (fibrosis) [5, 11]. In a review of more than 100 published studies [5], a multimodal approach was more common (66% of articles) than a single-modality approach (34% of articles) for characterizing fibrosis in nAMD, and CFP and FA was the most reported combination, likely because these methods were well established and accessible [5]. Areas of fibrosis that were delineated by polarization-sensitive OCT and/or multimodal imaging showed greater losses of microperimetric retinal sensitivity compared with regions deemed to have fibrosis based on unimodal imaging [22].

The commonly used established methods of detecting fibrosis, namely CFP and FA, are limited by inter-grader variability and a high dependency on image quality, which relies on optical media clarity and pupil dilation [5, 23]. In addition, FA carries risks associated with intravenous dye administration and there may be difficulty in distinguishing between staining and leakage [5, 24]. A further challenge with CFP is difficulty in differentiating fibrosis from other structures that have a similar appearance (e.g., atrophy, fibrin, lipids) [5]. The widespread availability of spectral-domain OCT is currently the preferred modality for imaging the macula. Its advantages are non-invasiveness and capacity to detect early, subtle forms of fibrosis [5]. OCT has similar limitations to CFP, with blood, fibrin, lipids, and vitelliform material, as well as fibrosis, appearing as hyperreflective regions [5, 25, 26]. However, the fluidic components of exudation can disappear over time with treatment and, when persistent, can be assumed to be fibrosis and, thus, the dynamic changes can be used as clues to the composition of the HRM [5, 25–27].

### Defining SHRM in nAMD

SHRM appears as an exudation into the subretinal space, serving as a key fibrosis biomarker or surrogate in OCT imaging [5, 26, 28]. SHRM may have a vascular component [29]. This vascular characteristic aligns with the development of early vascular networks that become more fibrotic over time due to endothelial–mesenchymal transition (EndMT) and epithelial–mesenchymal transition (EMT) [30, 31], and explains the difficulty in grading fibrosis, as leakage can be mistaken for staining [5]. Since collagen is birefringent, imaging modalities based on this property appear promising [32].

SHRM may be composed of MNV components, blood, lipids, vitelliform material, fibrin, and fibrotic tissue [5], and is common in eyes with nAMD, even following anti-VEGF therapy [26, 33].

The degree of reflectivity and definition of the borders may indicate responsiveness to anti‑VEGF therapy. For example, highly reflective materials with clear demarcation from the overlying retina do not respond to anti-VEGF therapy [5, 13]. Conversely, HRM with lower internal reflectivity described as undefined [25, 34] can respond to anti-VEGF therapy, as it is likely composed of resolvable elements [25, 34]. Although its composition is unclear, in treatment-naïve eyes undefined HRM correlates with fibrin on CFP [25].

## Mechanisms of fibrosis in nAMD

The pathogenesis of fibrosis in nAMD has been widely reviewed [7, 28, 30, 31, 35, 36]. In 1875, Pagenstecher described the disciform lesion, which was later found to be fibrotic when examined histologically [28, 37]. The fibrotic scar is characterized by increased deposition of extracellular matrix components, including collagen (predominantly types I and IV with small amounts of types III, V, and VI), laminin, fibronectin, and osteopontin (secreted-phosphorylated protein [SPP1]) [7, 30]. These molecules are generated mostly by myofibroblasts, which most likely arise via EMT and EndMT [7, 28, 38]. EMT may involve RPE cells, but other cell types also undergo mesenchymal transition, including macrophages, pericytes, and glial cells [28, 30, 36]. In surgically excised subretinal neovascularization, an expression gradient exists within the RPE from normal to myofibroblast, suggesting that the RPE is the major source of fibroblasts generated via EMT [35]. During angiogenesis, endothelial cell basement membranes break down while maintaining intracellular junctions, which allows cells to migrate in a chain formation [39]. The role of EndMT has been demonstrated in human endothelial cells and a mouse model of subretinal choroidal neovascularization (CNV) [40].

### EMT

EMT has three subtypes: type 1 EMT occurs in embryo development; type 2 EMT occurs during wound healing, tissue regeneration, and fibrosis; and type 3 EMT is associated with tumor invasion [31, 38]. Type 1 MNV lesions can develop fibrosis, but this tends to occur in the sub-RPE compartment [41]. EMT arises in RPE cells when cell–cell adhesions and apical-basal polarity are lost, enabling trans-differentiation and conversion into myofibroblasts, which are generally absent in adult tissues [7, 21, 28, 30, 31, 35, 36, 42–44]. During this process, RPE cells lose epithelial markers (E-cadherin and ZO-1) and acquire mesenchymal markers (N-cadherin, vimentin, and α smooth muscle actin [α-SMA] [7]). In addition, the cells undergo cytoskeletal reorganization with increased stress fiber formation and the replacement of intermediate filaments with vimentin, which enhances their motility and invasive capacity [45]. These changes are mirrored with activation of key transcription factors that suppress E-cadherin expression, including zinc finger protein SNAI1 (Snail), Slug, Twist, and zinc finger E-box-binding homeobox 1 [38, 45]. Nuclear localization of β-catenin, normally associated with the cell junctional complex, is a feature of EMT, and inhibiting β-catenin signaling prevents EMT [30]. This sequence of events indicates the early and pivotal role of E-cadherin loss within EMT.

### EndMT

EndMT is a similar process to EMT but involves the loss of endothelial markers (vascular endothelial‑cadherin, and platelet endothelial cell adhesion molecule 1) and the gain of mesenchymal markers (N-cadherin, vimentin, and α-SMA) [30]). However, a key difference between EMT and EndMT is the role of transforming growth factor (TGF)-β; although all three TGF-β isoforms can induce EMT, TGF-β2 is the main effector of EndMT [46]. EndMT may also be involved in angiogenesis. The endothelial sprouts at the leading edges of newly growing blood vessels form via EndMT and therefore possess mesenchymal properties: they are induced by TGF-β2, which is the predominant isoform found in the retina [30]. This may explain why angiogenesis generates fibrovascular networks with predominantly early vascular features that become more fibrotic over time. Furthermore, this highlights why fibrosis and wound healing share common characteristics.

The involvement of EndMT in the formation of fibrosis in CNV may also have implications for the treatment of nAMD due to VEGF involvement. A substantially reduced responsiveness to VEGF-A has been demonstrated in a study of primary human retinal endothelial cells with induced EndMT that were restored via suppression of EndMT [40]. Similarly, in a mouse model of spontaneous CNV, VEGF-A receptor antagonism includes further EndMT and fibrosis, indicating that anti-VEGF therapies may exacerbate EndMT-related fibrosis [40]. Further explanation for this relationship may be drawn from studies of proliferative diabetic retinopathy, in which fibrosis is linked to a shift in the ratio of VEGF to connective tissue growth factor (CTGF) in the eye [47]. CTGF is a profibrotic cytokine, and its expression in the RPE is induced by TGF-β [47–50]. Although CTGF levels were positively correlated with fibrosis formation, there was a negative correlation between the VEGF levels and fibrosis [47]. The ratio of CTGF:VEGF is a strong predictor of fibrosis and may trigger an angiofibrotic switch that drives fibrosis [47].

### Growth factors

RPE disruption (loss of RPE cell-cell contact) is necessary but insufficient for fibrosis formation; growth factors and inflammatory cytokines drive EMT [7, 35, 51]. These include TGF-β, fibroblast growth factor (FGF), epidermal growth factor, platelet-derived growth factor (PDGF), CTGF, tumor necrosis factor-α, and complement proteins, which may be released by RPE cells, infiltrating macrophages, and fibroblasts [7, 28, 30, 31, 35, 36]. These growth factors also act on myofibroblasts to promote profibrotic activities, such as cell proliferation, migration, and extracellular matrix remodeling through multiple signaling pathways involving p38 mitogen-activated protein kinase (MAPK), p44/p42 MAPK (ERK1/2), phosphatidylinositol-3 kinase (PI3K)/Akt (protein kinase B), and Smad [35].

## Clinical risk factors for fibrosis in nAMD

Several nAMD-related fibrosis risk factors exist. In the CATT studies, multivariate analysis showed an increased risk of scar development with type 2 MNV, larger lesion size, increased foveal retinal thickness, subretinal fluid, and presence of SHRM on OCT at baseline [7, 16, 19]. Potentially, as type 2 MNV lesions penetrate the RPE layer and grow into the subretinal space, they are more likely to contain damaged RPE cells, which may contribute to SHRM, and are therefore more likely to progress to fibrosis than type 1 MNV, which is confined to the sub-RPE space [7, 16, 35].

In a retrospective, single-center, observational study of 150 eyes of 144 patients with treatment-naïve nAMD, HRM presence at baseline was associated with an increased risk of macular scar development by 1 year compared with eyes with no HRM (χ^2^ 31.27; *p* < 0.001) [19]. Sub-RPE HRM location was strongly associated with macular scar development at 1 year (χ^2^ 82.1; *p* < 0.001), and both HRM thickness and width were identified as significant risk factors for the development of fibrotic scars (*p* < 0.001 and *p* = 0.02, respectively) but not non-fibrotic scars (*p* = 0.67 and *p* = 0.65, respectively) [19]. This study distinguished between undefined and well-defined HRM on OCT, hypothesizing that undefined HRM has a high fibrin and inflammatory infiltrate content, whereas well-defined HRM comprises neovascular complexes and/or pre-existing fibrous elements. Eyes with well-defined HRM had a higher risk of developing scarring than those with undefined HRM. It was suggested that well-defined HRM may be pre-existing scar tissue and that undefined HRM develops into scar tissue in some eyes [19].

Roberts et al. (2022) studied 45 eyes of 45 patients with nAMD over 1 year. Eight of the 45 eyes (18%) developed subretinal fibrosis during the study period [27]. There were no significant differences between eyes that did or did not develop subretinal fibrosis in any evaluated quantitative OCT angiography parameters or baseline microvascular measures; however, low baseline best-corrected visual acuity (BCVA) and presence of SHRM or intraretinal fluid were clearly identifiable indicators of increased risk of subretinal fibrosis [27].

SHRM, which may comprise fibrovascular or fibrocellular tissues and develop into fibrosis, separates the RPE and photoreceptor layers to cause photoreceptor dysfunction and vision loss [33]. A pooled treatment-agnostic *post hoc* analysis of the Phase III HAWK and HARRIER studies of brolucizumab 6 mg (*N* = 700) vs. aflibercept 2 mg (*N* = 696) reported better vision outcomes in eyes with lower SHRM thickness at 12 weeks post-loading and in eyes with less fluctuation in SHRM thickness to Week 96 [33]. Fluctuations in SHRM appeared to increase the likelihood of developing thicker SHRM over time. Likewise, analysis of the IVAN and CATT trials of anti-VEGF therapy in nAMD found that eyes that experienced more severe fluctuations in retinal thickness had worse BCVA and also had a higher risk of developing both fibrosis and macular atrophy [6].

## Impact of anti-VEGF therapy on fibrosis risk

Results of the ANCHOR and MARINA Phase III studies showed slower growth in the area of the CNV following anti-VEGF therapy than in eyes that had received verteporfin or sham injection, respectively [52, 53]. Here, we discuss the impact of the specific anti-VEGF agent, administration delays or extended intervals, and dosing or treatment regimen on fibrosis risk in nAMD (see Table 1).

**Table 1:** Summary of studies of anti-VEGF therapy and fibrosis risk in nAMD

Study name or authors	Study type/Phase	Patient population and sample size	Study group(s)	Primary or main outcome	Key secondary or other outcome(s)	Main fibrosis-related result(s)
ANCHOR	Phase III RCT	423 patients with predominantly classic nAMD	1:1:1 ratio to receive monthly IVT injections of ranibizumab (0.3 mg or 0.5 mg) plus sham verteporfin or monthly sham injections plus active verteporfin	Proportion of patients losing < 15 letters from baseline VA at 12 months	• Change in size of classic CNV • Change in size of leakage from CNV plus RPE staining • Change in size of CNV • Change in size of lesion	At 12 months, the area occupied by classic CNV decreased by a mean of 0.52 optic-disk area with ranibizumab 0.3 mg and 0.67 optic-disk area with ranibizumab 0.5 mg vs. a mean increase of 0.54 optic-disk area with verteporfin (*p* < 0.001 for each comparison)
MARINA	Phase III RCT	716 patients with nAMD with minimally classic or occult CNV	1:1:1 ratio to receive 24 monthly IVT injections of ranibizumab (0.3 mg or 0.5 mg) or sham injections	Proportion of patients losing < 15 letters from baseline VA at 12 months	• Mean changes from baseline in VA and Snellen equivalents at 12 and 24 months	Mean change from baseline in CNV growth and leakage was significantly better in both ranibizumab-treated groups vs. sham-injection group at 12 and 24 months (*p* < 0.001 for each comparison)
Angermann R, et al. (2022)	Retrospective comparative cohort study	648 eyes of 559 patients with nAMD	Treatment-naïve patients who received anti-VEGF therapy (aflibercept) comparing persistent vs. non-persistent patients (non-persistence defined as a visit-free interval > 6 months)	Changes in VA	Anatomic outcomes (CMT change, SRF and/or IRF change, fibrosis development)	• Significantly higher proportion of eyes developed foveal fibrosis in the nonpersistent group (*n* = 8 eyes; 5.0%) vs. the persistent group (*n* = 5 eyes; 1.2%; *p* = 0.013) • Four-fold higher risk of developing fovea-involving fibrosis in the non-persistent group
Nassisi M, et al. (2023)	Retrospective observational analysis	348 patients with nAMD and scheduled IVIs between March 1 and April 30, 2020	Non-adherent (missed ≥ 1 IVI; *n* = 215) and adherent groups (no missed IVIs; *n* = 83)	BCVA changes	• CRT • Type of MNV • Presence and location of fluid • Presence of cRORA • Presence of SHRM, further classified as fibrosis or SHE	• No significant difference between groups for fibrosis development at 6 months (*p* = 0.51)
Zhao X, et al. (2021)	Real-world retrospective review analysis	155 eyes of 130 patients with nAMD	Treatment delayed (missed follow-up visit more than 3 months due to COVID-19 pandemic) vs. non-delayed patients (treated/reviewed as scheduled)	• BCVA • CRT • SFCT • SRF • IRF • SRH • Massive SRH • VH • Foveal involvement • PED type		• Significant increase in proportion of patients with submacular scar in treatment-delay group (*p* = 0.043)
“Fight Retinal Blindness!” Registry	Real-world observational registry study	1559 eyes with nAMD and active disease despite anti-VEGF treatment every ≤ 6 weeks	Eyes treated with extended intervals (≥ 7 weeks) vs. non-extended intervals (≤ 6 weeks); eyes were further stratified into four groups by the mean interval over the following 6 months: <6 weeks, 7–9 weeks, 10–12 weeks and >12 weeks	Mean change in VA at 6 months by treatment interval group	• Proportion of eyes with ≥ 5-, ≥10- or ≥ 15-letter loss • Number of visits (including non-treatment visits, excluding visits for fellow eye if applicable) • Proportion of active visits	Mismatch with disease activity in the > 12-week interval group (72% of visits graded as active) and the ≤ 6-week interval group (70% of visits graded as active) and visual outcome may be due to development of macular atrophy or subretinal fibrosis in the longer-interval group
CATT	Prospective cohort study within an RCT	1059 eyes from patients enrolled in the CATT trial with nAMD and no scar on CFP or FA	Ranibizumab or bevacizumab treatment (any 1 of 3 dosing regimens – monthly, PRN, or monthly for 1 year followed by PRN)	Scar formation		• 480 of 1059 eyes (45.3%) developed scarring by 2 years • Baseline characteristics associated with increased scarring risk were classic vs. occult CNV, blocked fluorescence, foveal retinal thickness > 212 μm vs. < 120 μm, foveal subretinal tissue complex thickness > 275 μm vs. ≤ 75 μm, foveal SRF vs. no SRF, and SHRM vs. no SHRM • Baseline RPE elevation was associated with lower risk of scarring vs. no RPE elevation • Drug type, dose regimen, and genotype had no statistically significant effect on scarring • Fibrotic scars developed in 24.7% of eyes vs. non-fibrotic scars in 20.6% of eyes • Baseline risk factors for both scar types were similar except eyes with larger lesions or VA < 20/40 were more likely to develop fibrotic scars
Bloch SB, et al. (2013)	Retrospective observational case series	197 eyes of 197 patients with nAMD and no subfoveal fibrosis	Treatment-naïve patients who received anti-VEGF therapy (PRN ranibizumab)	Subfoveal fibrosis at ≤ 24 months	• VA • CNV area • Type of lesion	• Interval > 14 days between nAMD diagnosis and anti-VEGF initiation conferred a 2-fold risk of fibrosis at follow-up vs. interval < 14 days (HR 2.24; 95% CI 1.28 to 3.94; *p* = 0.005) • Eyes that developed prominent fibrosis or fibrosis with foveal atrophy lost 8.5 more ETDRS letters (95% CI, -1.0 to -15.9; *p* = 0.0242) and 10.3 more ETDRS letters (95% CI, -4.0 to -16.5; *p* = 0.0012) vs. eyes that did not develop fibrosis, respectively
HAWK + HARRIER pooled analysis	Pooled post hoc analysis of two Phase III trials	OCT images from 1396 patients with nAMD treated with anti-VEGF therapy	700 brolucizumab-treated patients, 696 aflibercept-treated patients	Maximum SHRM thickness across 96 weeks	Effect of Week 12 SHRM thickness and SHRM variability on BCVA regardless of treatment	Numerically higher percentage reductions in SHRM thickness (a known risk factor for fibrosis), from baseline to Week 96 with brolucizumab vs. aflibercept (54.4% vs. 47.6%)
Lee J, et al. (2022)	Retrospective consecutive case series	72 patients with 72 eyes with nAMD who improved on initial monthly injections but rapidly worsened after switching to bi-monthly maintenance injections	Monthly alternating aflibercept and bevacizumab (MAAB) injections or bi-monthly aflibercept (BiA) injections	• BCVA change during 24 months; CRT change at 24 months and baseline factors likely to affect worsening after changing to aflibercept bi-monthly were also evaluated	• Retinal fluid status in the maintenance phase • Visual and anatomical changes by nAMD subtype and prior treatments	Incidence of disciform scar development (6 vs. 14, *p* = 0.710) was not different between the MAAB and BiA groups, respectively
Muftuoglu IK, et al. (2017)	Retrospective single-center consecutive cases review	43 eyes of 39 patients with nAMD who had failed bevacizumab or ranibizumab treatment, and who had responded poorly to Q8W aflibercept	Aflibercept Q4W PRN	MRT, CMT, maximum PED elevation and BCVA were compared with baseline when aflibercept Q4W was initiated		At 1 year, 12 eyes (28%) had some subretinal scarring, and 5 eyes (11.6%) had evidence of atrophy
Adrean SD, et al. (2021)	Retrospective single-center retina-only clinic database study	143 eyes with nAMD	88/143 eyes with nAMD from patients that completed a treat-and-extend anti-VEGF protocol	• Vision • Injection quantity • Initial lesion size • Final anatomic status		• Twenty-five of 88 eyes (28.4%) developed fibrovascular scarring • Pre-, post- and final vision for eyes with fibrovascular scarring were 22.4 letters (20/400 + 2) vs. 11.6 letters (20/640, *p* = 0.0351) vs. 11.0 letters (20/640 + 1, *p* = 0.0226), respectively
Barikian A, et al. (2015)	Single-center prospective randomized pilot study	90 eyes from 90 treatment-naïve patients with nAMD	1:1:1 randomization to Q2W for 3 consecutive injections, Q4W for 3 consecutive injections, or immediate PRN after the first injection	Initial fluid-free interval after induction	• Mean BCVA change • Mean CRT change	Six eyes in the Q2W induction group developed subretinal fibrosis vs. no eyes in the other 2 groups (*p* = 0.003)

BCVA, best-corrected visual acuity; BiA, bi-monthly aflibercept; CFP, color fundus photography; CI, confidence interval; CMT, central macular thickness; CNV, choroidal neovascularization; cRORA, complete RPE and outer retinal atrophy; CRT, central retinal thickness; ETDRS, Early Treatment Diabetic Retinopathy Study; FA, fluorescein angiography; HR, hazard ratio; IRF, intraretinal fluid; IVI, intravitreal injection; IVT, intravitreal; MAAB, alternating aflibercept and bevacizumab; MNV, macular neovascularization; MRT, maximum retinal thickness; nAMD, neovascular age-related macular degeneration; OCT, optical coherence tomography; PED, pigment epithelium detachment; PRN, pro re nata; Q2W, every 2 weeks; Q4W, every 4 weeks; Q8W, every 8 weeks; RCT, randomized controlled trial; RPE, retinal pigment epithelium; SFCT, subfoveal choroidal thickness; SHE, subretinal hyperreflective exudation; SHRM, subretinal hyper-reflective material; SRF, subretinal fluid; SRH, subretinal hemorrhage; VA, visual acuity; VEGF, vascular endothelial growth factor; VH, vitreous hemorrhage

In a real-world cohort of 648 treatment-naïve eyes of 559 patients, subfoveal fibrosis developed in a lower proportion of eyes of patients who persisted with anti-VEGF therapy (1.2%, *n* = 5/405 eyes; *p* = 0.013) vs. non-compliant patients (defined as a visit-free interval of more than 6 months; 5.0%, *n* = 8/161 eyes); age-adjusted analysis revealed a four-fold higher risk of developing a fovea-involving fibrosis in the non-compliant group [54]. The COVID-19 pandemic resulted in delays in anti-VEGF therapy that impacted the risk of fibrosis. In a retrospective analysis to investigate delayed anti-VEGF therapy during the pandemic, patients were categorized as non-adherent if at least one intravitreal (IVT) injection was missed (*n* = 215) between March 1st and April 30th, 2020 and adherent if their treatment schedule was followed (*n* = 83); no significant difference was found between the groups for incidence of fibrosis [55]. A similar retrospective study of 155 eyes of 130 patients with nAMD defined delay as missing regular follow-up visits for more than 3 months. In this analysis, the proportion of patients presenting with submacular scarring was significantly greater in the delayed-treatment group compared with the non-delayed group (*p* < 0.05) [56]. In the real-world “Fight Retinal Blindness!” registry of patients with nAMD undergoing unplanned treatment interval extensions before the COVID-19 pandemic, a significant short-term loss in VA was evident in eyes of patients for whom the re-treatment interval was extended to more than 12 weeks compared with the non-extended group of no more than 6 weeks (*p* = 0.03). Differences in visual outcomes between the two groups may be due to the development of poor prognostic features, such as subretinal fibrosis, in the extended-interval group, limiting patients’ visual improvements [57].

However, long-term results from cohort studies (e.g., CATT) revealed that fibrosis remains a problem despite anti-VEGF therapy [5]; the incidence of fibrosis is highest in the first year of treatment, reducing over time [11].

Suboptimal anti-VEGF therapy is a risk factor for development or worsening of fibrosis after treatment initiation [12, 55, 56]. In a retrospective, observational case series of 197 treatment-naïve eyes of 197 patients, an interval of at least 14 days between diagnosing nAMD and initiating anti-VEGF therapy conferred a two-fold risk of fibrosis at follow-up compared with an interval of less than 14 days (hazard ratio [HR] 2.24; 95% CI 1.28–3.94; *p* = 0.005) [58]. Furthermore, increased fluctuations in retinal thickness are a risk factor for fibrosis [6], and extending treatment intervals will likely lead to greater fluctuations in retinal thickness than continuous monthly injections [59].

### Anti-VEGF agent type

To date, no obvious association has been identified between different currently available anti-VEGF agents and the development of fibrosis [4, 16, 19, 60, 61]. For example, in the CATT studies, the frequency of fibrotic scar development was not influenced by ranibizumab or bevacizumab treatment (adjusted HR 1.2; 95% CI 0.96–1.4) [16]. However, a recent pooled *post hoc* analysis of the HAWK and HARRIER trials showed that brolucizumab was associated with numerically greater reductions in SHRM thickness, a known risk factor for fibrosis, from baseline to Week 96 vs. aflibercept (54.4% vs. 47.6%) [33].

### Treatment regimen

In the CATT studies, the dosing regimen (*pro re nata* [PRN] vs. switch from monthly to PRN) had no statistically significant association with the development of fibrosis (adjusted HR 0.9; 95% CI 0.8–1.1) [16]. A retrospective, consecutive case series of 72 eyes of 72 patients with nAMD found no difference in scarring development at 24 months between monthly alternating injections of aflibercept and bevacizumab compared with bimonthly aflibercept (6 vs. 14 eyes; *p* = 0.710) [60]. In a study of 43 eyes of 39 patients with nAMD in whom prior anti-VEGF therapy had failed, treatment with high-frequency aflibercept injections (PRN, every 4 weeks [Q4W]) showed evidence of subretinal scar tissue formation in 12 eyes (27.9%) at 1 year [62]. A similar proportion of eyes (28.4%; *n* = 25/143) in patients with nAMD developed fibrovascular scarring in a study using a treat-extend-stop protocol of anti-VEGF therapy (at least 3 monthly injections followed by 1- to 2-week injection interval extensions to 12 weeks) [63]. In a prospective, single-center study, patients with treatment-naïve nAMD received one of three bevacizumab induction sequences: biweekly, Q4W, or immediate PRN after the first injection (30 eyes per group): 6 eyes (20%) in the biweekly induction group developed subretinal fibrosis compared with no eyes in the other two groups (*p* = 0.003) [64]. This evidence suggests that PRN or monthly dosing regimens pose little risk of fibrosis, although this risk increases with more regular anti-VEGF injections.

## Impact of early detection of nAMD on fibrosis

Macular fibrosis is irreversible after onset and associated with permanent vision loss; therefore, early detection is essential in ensuring the best possible outcome for patients. Since fibrosis in nAMD develops from the existing neovascular membrane, the identification and regression of smaller MNV lesions and/or the prevention of neovascular complex expansion at the very early stages of nAMD may reduce the likelihood of fibrosis occurring [28].

Early detection of nAMD reduced the prevalence of SHRM, improved VA, and maintained quality of life vs. conventional diagnosis [65]. FASBAT was a 2-year, prospective, multicenter, observational study nested within the EDNA (Early Detection of Neovascular AMD) trial [65]. A multimodal approach was used for early detection of nAMD in the second eyes of patients undergoing routine care for nAMD in their first eye [65]. Among 431 patients in FASBAT, 100 experienced conversion to nAMD in the second eye after a mean of 18.9 months [65]. The prevalence of SHRM on OCT was lower at early diagnosis in the second eye (77.2% [*n* = 71] at conversion and 80.5% [*n* = 70] at 24.9 months post-conversion) compared with conventional diagnosis in the first eye (93.0% [*n* = 93] at baseline and 92.4% [*n* = 85] at 18.9 months post-conversion) [65]. Mean VA was 18 letters better at the time of early diagnosis in the second eye compared with conventional diagnosis in the first eye (72.9 [standard deviation (SD) 8.1] vs. 55.6 [SD 15.7] letters) [65]. Mean VA remained better in the second eye 24.9 months post-conversion, at 69.5 (SD 14.0) letters vs. 59.7 (SD 20.5) letters at a similar matched time point in the first eye (18.9 months) [65]. Over time, the mean composite quality of life scores increased, significantly correlating with VA for the second eye only 24.9 months post-conversion (*p* < 0.005) [65]. Early diagnosis may result in reduced fibrosis and fibrin, and an identifiable neovascular membrane [65]. These data highlight the substantial need for early detection and treatment of nAMD before permanent structural damage, such as fibrosis, occurs.

## Future directions

**Fig. 2:**
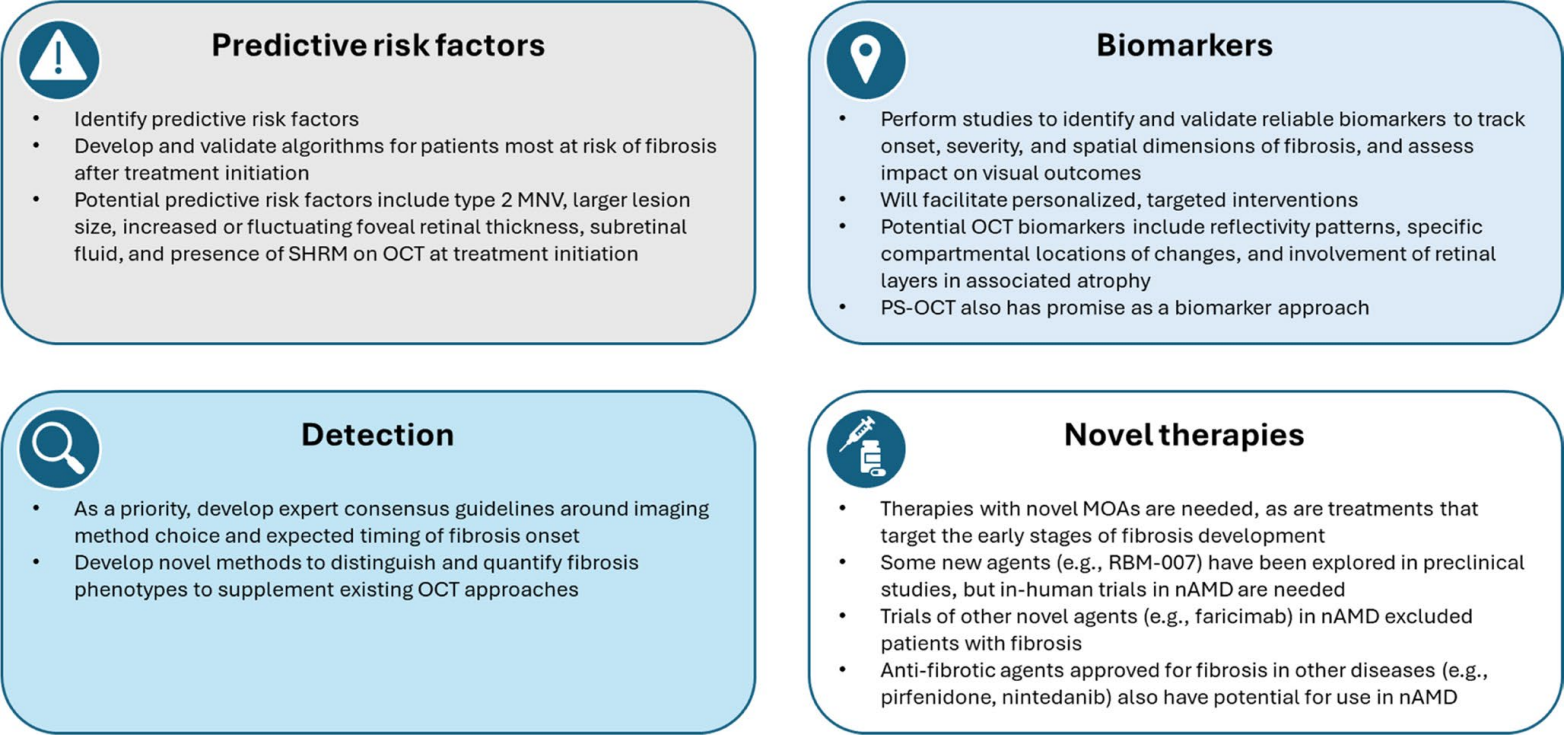
Key areas for future directions for fibrosis management in nAMD. MNV, macular neovascularization; MOA, mode of action; nAMD, neovascular age-related macular degeneration; OCT, optical coherence tomography; PS-OCT, polarization-sensitive optical coherence tomography; SHRM, subretinal hyperreflective material

In this section, we discuss future directions for fibrosis management in nAMD across a range of topics, which are also summarized in Fig. 2.

### Predictive risk factors for fibrosis

Despite regular anti-VEGF therapy, approximately one-third of patients with subretinal fibrosis will experience irreversible vision loss [28]. Currently, predicting which patients will develop fibrosis during anti-VEGF therapy is difficult, and the combined CATT and IVAN analysis showed that patients experience progression to atrophy and/or fibrosis even with optimal therapeutic management of nAMD [6]. There are several reasons why fibrosis can develop during therapy, including suboptimal management, poor patient compliance, and delays in re-treatment [66]. Identification of key risk factors for fibrosis has important implications for maintenance of functional visual gains and requires continual monitoring to ensure timely re-treatment, placing a high financial and time burden on both patients and caregivers [66]. It has been identified that presence of type 2 MNV, larger lesion size, increased or variability of foveal retinal thickness, subretinal fluid, and the presence of SHRM on OCT at treatment initiation were risk factors for retinal fibrosis [6, 7, 16, 19]. Findings such as these could be used as the basis for developing a predictive risk algorithm that might help identify patients who are most at risk of developing fibrosis and, therefore, require more intensive monitoring.

### Biomarkers of fibrosis

Given the dynamic nature of fibrosis, longitudinal studies are needed to identify and validate reliable biomarkers to effectively track the onset, severity, and spatial dimensions of fibrosis, and assess its impact on visual outcomes [5]. Visual outcomes would be modified by the proximity of the structural changes to the fovea and the extent of distribution within the affected area [5]. Promising potential OCT biomarkers of fibrosis include patterns of reflectivity, the specific compartmental locations of changes (e.g., subretinal, beneath the RPE, a combination of both), and the involvement of retinal layers in associated atrophy [5]. Comprehensive clinicopathological correlation studies are also needed to establish the clinical significance and inter-relationships of OCT-derived biomarkers, thereby enhancing the diagnostic and prognostic capabilities for managing fibrosis in nAMD [5]. Another promising approach for use as a biomarker of fibrosis in nAMD is use of polarization-sensitive OCT (PS-OCT). A high-resolution non-invasive imaging method, PS-OCT detects changes in light polarization state following its interaction with tissue, facilitating higher contrast separation of different tissue layers [5]. nAMD-related fibrosis uniformly displays birefringence on PS-OCT, while related properties such as optic axis uniformity and local-phase retardation have also been reported [5]. PS-OCT therefore has the potential to differentiate between fibrosis and other retinal hyper-reflective features (e.g., macular neovascularization, blood, lipids, fibrin) and also from the highly reflective RPE [5]. Validation of such biomarkers is necessary to allow diagnosis and better management of nAMD and could facilitate personalized, targeted interventions to improve patient outcomes by enabling earlier and more precise treatment adjustments.

### Detection of fibrosis

As mentioned, consensus regarding the type of imaging modalities and time point when identification of fibrosis should occur is lacking. Expert consensus criteria that are objective, highly reproducible, and capable of detecting both early and subtle forms of fibrosis [5] are needed, and consensus on these criteria should be a priority for the near future. Other important future considerations for detecting fibrosis include the development of a classification system that can clearly distinguish the different phenotypes of fibrosis along with robust validated methods for quantifying fibrosis [5]. Systematic reviews have shown that the detection of fibrosis is influenced by imaging modality [5]. Also an agreement study has reported varying sensitivity and specifity for *en face* technologies of color and FA vs. OCT, which is also influenced by the location of fibrosis, whether subretinal or sub-RPE [67]. As imaging technologies continue to improve, other novel approaches may enable easier detection of fibrosis in the context of nAMD.

### Developing new therapies for fibrosis

Effective new strategies are needed to prevent the development of fibrosis in nAMD, notably those with novel modes of action that can inactivate pathological neovascularization and prevent EMT and EndMT and collagen deposition. CNV mouse models have demonstrated sustained prevention of scarring with the angiopoietin-2/VEGF-A bispecific antibody faricimab [68]. Faricimab 6.0 mg, administered at up to 16-week intervals, showed vision benefits similar to those seen with aflibercept 2.0 mg at 8-week intervals in two Phase III trials [69]. However, the efficacy of faricimab in preventing fibrosis has not been assessed because Phase III clinical trial eligibility criteria often exclude eyes of patients with nAMD with fibrosis who are suspected of having long-standing disease or prior treatment for nAMD [69]. Trial durations, often of 2 years, may also be insufficient to detect longer-term outcomes. In addition, some post-licensing or comparative effectiveness trials may mandate that participants are responsive to anti-VEGF therapy, resulting in the enrollment of study eyes with type 1 CNV that respond well to treatment. Hence, data obtained in clinical trials do not reflect real-world outcomes. To improve the translation of clinical trial results to the real world, the overall patient population and the intensity of the proposed treatment schedule should be considered when designing future trials.

There is also a requirement for direct therapeutic strategies in nAMD, including anti-fibrotic agents. FGF2 is implicated in the pathophysiology of both angiogenesis and fibrosis [70]. In a CNV mouse model, the anti-FGF2 ribonucleic acid aptamer RBM-007 reduced new blood vessel formation and subretinal fibrosis [70]. RBM-007 has also been explored in a Phase I/IIa clinical study (SUSHI) in patients whose disease was unresponsive to prior anti-VEGF therapy, where it showed efficacy vs. anti-VEGF therapy for the endpoints of BCVA and central subfield thickness (CST) [71]. In the subsequent Phase II TOFU study of RBM-007 with or without aflibercept vs. aflibercept alone in patients with nAMD and poor response to existing anti-VEGF treatment, SHRM change at Week 16 was included as a secondary endpoint [72]. However, in the RBM-007–alone group, only 10.6% of patients showed improvement in SHRM, while the RBM-007 plus aflibercept and aflibercept-alone group outcomes were similar (28.6% and 31.0% of patients, respectively, showed improvement in SHRM) [73].

Anti-fibrotic agents are effective in other disease areas. Pirfenidone was approved in 2011 by the European Medicines Agency and in 2014 by the US Food and Drug Administration for the treatment of adults with idiopathic pulmonary fibrosis (IPF) [74, 75]. Pirfenidone is a type of immunosuppressant with antifibrotic and anti-inflammatory properties. Although its mechanism of action is not fully established, it prevents fibroblast proliferation, fibrosis-associated protein and cytokine production, and increased extracellular matrix biosynthesis and accumulation in response to cytokine growth factors (e.g., TGF-β, PDGF) [74]. Injection and topical administration of pirfenidone in rabbit models of glaucoma drainage implant surgery improved both fibrosis and trabeculectomy bleb survival [76, 77]. In a preclinical study in which retinal injury was induced in rabbit eyes to mimic proliferative vitreoretinopathy arising from ocular injury, a significant decrease in proliferative vitreoretinopathy score was observed in eyes treated with pirfenidone compared with untreated control eyes (0.78 vs. 2.33, respectively; *p* = 0.0003) [78]. Pirfenidone also reduced expression of collagen-I, α-SMA, and TGF-β vs. control eyes [78]. More recently, in a laser‑induced mouse model of CNV, areas of CNV, areas of CNV leakage, and expression of TGF-β2 and VEGF were reduced in eyes treated with pirfenidone compared with control eyes 1 week after photocoagulation [79]. On Day 28, no abnormalities in neural retinal layers were observed in pirfenidone-treated mice [79]. In a similar study of pirfenidone administered via drinking water, scar tissue formation and expression of extracellular matrix proteins and inflammatory markers were reduced compared with animals who received water alone [80]. However, pirfenidone treatment did not result in regeneration of the photoreceptor layer in these mice [80]. Pirfenidone has been shown to reduce TGF-β2-induced neovascularization and fibrosis by suppressing the nuclear factor kappa B/Snail signaling pathway in ARPE-19 cells (a spontaneously arising RPE cell line) [81]. These findings suggest that pirfenidone may be a promising therapy for fibrosis in nAMD. Administration of pirfenidone to the eye in polyester nanoparticles, polyurethane nanocapsules, and soft contact lenses has been assessed *in vitro* and *in vivo*, showing promise as ocular drug delivery systems [82–84].

Data from the Phase II BETTER trial of ISTH0036, a selective transforming growth factor-β2–blocking antisense oligonucleotide being investigated for the treatment of retinal fibrosis in patients with nAMD and diabetic macular edema (DME), were presented at the 2025 ARVO Annual Congress [85]. IVT injections of ISTH0036 every 8 weeks (Q8W) were well tolerated and not only stabilized or improved BCVA as well as reduced CST across all groups, but also significantly decreased HRM volume in eyes with nAMD and fibrosis-associated HRM [85]. In contrast, HRM volume increased in eyes treated with anti-VEGF therapy [85]. These findings highlight the potential for this therapeutic approach in nAMD [85].

Multiple tyrosine kinase inhibitors (TKIs) are also under investigation for the treatment of nAMD, including a bioerodible vorolanib IVT insert, injectable suspension and IVT hydrogel formulations of axitinib, and an IVT depot formulation of sunitinib. Two Phase III trials (LUGANO and LUCIA) of vorolanib in nAMD were initiated in 2024 [86], as was the Phase III SOL-1 study of the AXPAXLI implant formulation of axitinib [87]. Data are available from the Phase I/IIa OASIS trial of the injectable suspension axitinib formulation [88] and the Phase IIb ALTISSIMO trial of sunitinib in nAMD [89]. In OASIS, up to 1.0 mg of axitinib suspension was well tolerated, with stable mean VA and mean CST, while most patients did not require aflibercept therapy during a 6-month follow-up period [89]. In ALTISSIMO, median time to the primary endpoint of first rescue medication was 5 months in patients who received 1-mg doses of axitinib suspension at baseline and 6 months, and 4 months in patients receiving a 2-mg dose at baseline and a 1-mg dose at 6 months [88]. Promising anti-fibrotic effects of these TKIs have been demonstrated in several studies. In a mouse model of retinal detachment, vorolanib reduced fibrosis in the outer nuclear layer of the retina [90], while in cell culture and mouse models of liver fibrosis, axitinib was able to reduce progression of fibrosis by restoring mitochondrial function [91]. Similarly, sunitinib has shown anti-fibrotic effects in a bleomycin-induced mouse model of pulmonary fibrosis [92], as well as a decrease in expression of markers of fibrosis in fibrotic livers [93]. Together, these findings highlight the therapeutic potential of these agents for reducing or preventing nAMD-related fibrosis.

## Conclusions

In nAMD, development of MNV lesions causes chronic damage and inflammation resulting in fibrosis. Development of fibrotic scarring disrupts the highly organized anatomical layers and tightly coordinated cellular interactions in the retina, resulting in profound and irreversible visual impairment.

Optimal management of nAMD can reduce the likelihood of fibrosis development. How anti-VEGF agents are used with respect to specific treatment regimens, treatment delays, and extended administration intervals between visits can affect the development of fibrosis in nAMD. However, most published research focuses on Caucasian populations, and differences in patient and disease characteristics may affect the development of fibrosis and response to treatment.

Early detection and treatment of nAMD, before worsening of fibrosis, is crucial to preserve VA gains. Understanding the drivers and risk factors for fibrosis development is also critical to limit its effects and maintain early VA gains in the long term. Patients with clinical risk factors and ocular anatomical biomarkers of risk for the development of fibrosis should be closely monitored. An expert consensus on definitions, grading criteria, and diagnostic process for fibrosis is urgently needed, as the presence of fibrosis remains a limiting factor of VA. There is also a substantial need for direct therapeutic strategies for fibrosis in nAMD, including anti-fibrotic agents.
